# Circulating Small Extracellular Vesicles Profiling and Thrombin Generation as Potential Markers of Thrombotic Risk in Glioma Patients

**DOI:** 10.3389/fcvm.2022.789937

**Published:** 2022-06-23

**Authors:** Olga Melnichnikova, Yulia Zhilenkova, Olga Sirotkina, Ekaterina Zolotova, Konstantin Pishchulov, Malik Tastanbekov, Artem Paltsev, Maria Simakova

**Affiliations:** ^1^Personalized Medicine Centre, Almazov National Medical Research Centre, Saint Petersburg, Russia; ^2^Department of Laboratory Medicine and Genetics, Almazov National Medical Research Centre, Saint Petersburg, Russia; ^3^Department of Neurosurgery, Almazov National Medical Research Centre, Saint Petersburg, Russia

**Keywords:** microvescicle, glioma, thrombin generation, venous thromboembolism (VTE), D-dimer (DD)

## Abstract

**Introduction:**

Patients with glioma (GM) are at a high risk of venous thromboembolism (VTE). The role of microvesiculation in the cancer-associated thrombosis mechanisms has been previously demonstrated. This study aimed to evaluate the relative abundance of extracellular vesicles (EVs) and thrombin generation (TG) in combination with standard laboratory tests in patients with newly diagnosed GM as potential prognostic markers in VTE.

**Materials and Methods:**

In the present study, 11 patients with newly diagnosed GM and 10 healthy volunteers were analyzed. EVs were counted and their cellular origin was determined (CytoFlex B4-R2-V2, Beckman Coulter, United States), as well as thrombin generation test (TGT) (Diagnostica Stago SAS, France) was performed.

**Results:**

In patients with GM, the relative abundance of the CD41 + EVs (platelet-derived)—and CD105 + EVs (endothelial-derived) was significantly higher than in the control group (44.3 [40.5; 52.4] vs. 27.2 [22.9; 31.0]%, *p* = 0.002, and 5.4 [4.8; 7.8] vs. 1.9 [1.5; 2.8]%, *p* = 0.0003, respectively). The D-dimer level was higher in patients with GM compared with the control group (0.46 [0.38; 1.85] vs. 0.36 [0.27; 0.40] μg/ml FEU, *p* = 0.03, respectively). There was a trend toward an increase in the peak thrombin and velocity index (VI) in the GM group (*p* = 0.06). During the follow-up period, two patients (18%) developed thrombosis, had tumor sizes of more than 5 cm, thrombocytopenia, increased VI, and D-dimer.

**Conclusion:**

Analysis of platelet-derived EVs, platelet count, and TGT in combination with D-dimer assessment could improve the stratification of patients prone to VTE, which needs to be confirmed in a larger sample.

## Introduction

Patients with active cancer, such as brain tumors, have a much higher venous thromboembolism (VTE) risk than the general population (1, 2). However, the molecular and cellular mechanisms of cancer-associated thrombosis are complex and unclear. Cancer cells directly or indirectly activate the hemostasis system resulting in a hypercoagulable state. Tissue factor (TF), which is exposed to damaged tissues and can be expressed by tumor cells, may play a critical role in the prothrombotic profile as it is a powerful trigger for blood clotting (3).

In addition, TF can be expressed in the vessel wall and on the surface by cell-derived extracellular vesicles (EVs) (4). EVs are secreted by all cell types and act as mediators of intercellular communication through a specific load from the parent cell (microRNA, proteins, and lipids) (5). EVs have received particular attention due to their presence in circulating blood, involvement in cancer-associated thrombosis, and their possible connection with vesiculation processes and glioma (GM) progression. EVs have procoagulant activity using TF or can carry coagulation enzymes, acting both regularly and systemically (6). A study by Sartori et al. (7) showed the relationship between EVs and an increased risk of thrombosis in patients with GM.

It is known that TF and thrombin act as key mediators in the establishment of the pathophysiological link between cancer and thrombosis (8). Thrombin, in addition to its central role in the hemostatic system, is involved in cell proliferation, migration, differentiation, angiogenesis, and vascular tone modulation, as well as in oncogenesis (9). However, the role of thrombin in the brain has not yet been fully studied. The central nervous system is the only place of extra-hepatic thrombin expression, where it is involved in the development, protection, and regeneration of the brain, and could locally affect the processes of tumor formation and thrombosis (10).

Thus, further study of the thrombosis mechanisms in primary brain tumors will improve the risk stratification by using an individual approach based on the assessment of new biomarkers. In this pilot study, we analyzed EVs of various origins and results from the thrombin generation test (TGT) as markers of hypercoagulation in patients with GM.

## Materials and Methods

### Patients

Patients with newly diagnosed GM (5 men and 6 women) with a median age of 61 years [47; 64] who consecutively underwent surgery at the Neurosurgery Department of Almazov National Medical Research Centre in May 2021 were prospectively assessed. The inclusion criteria were: age 18 years or over, brain magnetic resonance images (MRI), and histological findings compatible with GM. The exclusion criteria were: pregnancy, severe liver or renal failure, and patients with tumor progression. All patients were treated with dexamethasone for cerebral edema with an average daily dose of 6 mg [4; 8]. No patient received antithrombotic treatment during blood sampling: anticoagulants were interrupted 24 h before the surgery, and antiplatelet therapy was interrupted 5 days before the surgery according to the local protocol. Pulmonary embolism was clinically suspected based on the Wells pretest probability score and the D-dimer levels recorded for the first 7–14 postoperative days. Deep-vein thrombosis was confirmed by compression ultrasound. As controls, ten healthy age- and sex-matched volunteers, without personal and familial history of thrombosis, not taking drugs affecting the coagulation system (anticoagulants and antiplatelet therapy), were tested as controls: five men and five women, with a median age of 48 years [44; 52]. The study protocol was approved by the Regional Ethics Committee (No. 0603-21). Written informed consent in accordance with the Declaration of Helsinki was obtained from all participants before their inclusion in the study.

### Blood Sampling and Laboratory Assays

Blood samples were drawn from each patient in vacuum tubes (VACUETTE), with K_2_EDTA for preparing plasma for analysis of EVs and with 3.2% sodium citrate for coagulation assays, followed by the International Society of Extracellular Vesicles (ISEV) and International Society for Thrombosis and Hemostasis (ISTH) for pre-analytical parameters (11, 12). The blood samples were tested for complete blood count using an automated 5-Diff blood counter (Cell-Dyn Ruby Abbott, United States), C-reactive protein levels (Cobas 2000, Roche Diagnostics, Switzerland), and coagulation assays [activated partial thromboplastin time (APPT), prothrombin time (PT), and fibrinogen levels] (STA-compact, Diagnostica Stago SAS, France). D-dimer levels were measured by immunoturbidimetric assays using commercial reagents (Diagnostica Stago SAS, France).

Blood samples for EVs measurement were double centrifuged at 2,200 × g for 15 min according to ISTH protocol (13). For TGT, centrifugation at 2,000 g for 10 min and then 10,000 g for 20 min was performed. The aliquots were stored at –80°C.

#### Isolation of Extracellular Vesicles

Isolation and identification of EVs were performed using the Exo-FACS kit (HansaBioMed Life Sciences, Estonia) according to the manufacturer’s recommendations. The kit consisted of ExoPrep reagent (polymer solution)—for chemical precipitation of EVs, latex FACS-Beads—for the overall capture of pre-isolated EVs, and antibodies against tetraspanin (CD9 AF488)—for inner membrane marker detection on the surface of EVs.

The plasma samples were additionally centrifugated at 10,000 × g for 30 min to remove cells and debris and then were incubated with ExoPrep reagent followed by centrifugation at 10,000 × g. Isolated EVs were incubated with FACS-Beads. Complex EVs-Beads were centrifuged and then double washed in the sample buffer (reagent from the kit, diluted in PBS × 5).

#### Nanoparticle Tracking Analysis

The size, homogeneity, and concentration of EVs in the obtained preparations were assessed using nanoparticle tracking analysis (NTA) conducted with the NanoSight^®^ LM10 (Malvern Instruments, United Kingdom) analyzer equipped with a blue laser (45 mW at 488 nm) and a C11440-5B camera (Hamamatsu photonics K. K., Hamamatsu, Japan). The NTA 2.3 software was used to record and analyze the obtained results. When analyzing records lasting 60 s, the following parameters were evaluated: the average hydrodynamic diameter (nm) and the concentration of microvesicles in suspension (particles/ml). As a result, a population of generally homogeneous small particles of 113 ± 43 nm (mean ± SD) was observed in the samples (Supplementary Figure 1).

#### Flow Cytometry

Extracellular vesicle analysis was performed using a CytoFLEX B4-R2-V2 (Beckman Coulter, United States) flow cytometer. The data obtained were analyzed using the CytoExpert v.2.3 (Beckman Coulter, United States).

Phenotyping of cell-derived EVs was carried out using the following monoclonal antibodies: CD41-PE/Cy7—platelet specific markers, CD235a-PE—erythrocyte, CD45-PC7—leukocyte, and CD105-PE—endotheliocyte (Biolegend, United States). Staining was performed after incubation with primary and secondary (AF488) antibodies against CD9, according to the manufacturer’s recommendations.

All control samples (Supplementary Figures 2,3), detergent effectiveness treatments (Supplementary Figure 4), and gaiting strategy (Supplementary Figure 5) were performed in accordance with the previously published requirements (14).

The expressions of platelet, erythrocyte, leukocyte, and endothelial cells markers on the EVs surface were assessed as the percentage of positive EVs (CD9 +) labeled with CD41, CD235a, CD45, and CD105, respectively (the relative abundance of the EVs, positive by each marker) (15). The representative flow cytometry plots of patient and donor are presented in the Supplementary Figure 6.

#### Thrombin Generation Test

In all study groups, thrombin generation (TG) was performed using the calibrated automated thrombinography method as described by Hemker et al. (16). To trigger the reagent (REF 86193), the TG reaction was used, which contained a medium concentration mixture of TF and procoagulant phospholipids. Thrombin-specific substrate (REF 86197) was labeled with fluorogenic aminomethylcoumarin (Z-Gly-Gly-Arg-AMC) (Diagnostica Stago SAS, France). A mixture of substrate and calcium-containing buffer was automatically added to the well with the reagent (REF 86193) and the patient’s plasma. The signal was recorded using a Fluoroskan Ascent^®^ device (ThermoFisher Scientific, United States). The test results were calibrated against the activity of the thrombin calibrator (REF 86192). Five parameters of TGT were calculated: maximum concentration of thrombin in the sample (Peakthr.), nmol; area under the thrombin formation curve–endogenous thrombin potential (ETP), nmol/min; coagulation initiation time (LT), min; time to peak thrombin concentration (ttPeak), min. The rate of thrombin formation [velocity index (VI), nmol/min] was calculated using the following formula (17): thrombin VI = Peakthr./(ttPeak-LT).

#### Statistics

The obtained data were processed using the SPSS Statistics software (version 21.0). Quantitative data were described using the median, 25th, and 75th percentiles (Me [Q_1_-Q_3_]). The analysis of qualitative data was carried out using the Pearson X2 test or Fisher’s exact test. A comparison of two independent variables was performed with the Mann–Whitney test. When the number of groups was greater than two, the Kruskal–Wallis test was applied. The Spearman rank correlation coefficient was used to estimate the relationship between quantitative variables. Differences were considered significant at *p* < 0.05.

## Results

### Clinical Data

In this study, 11 consecutive patients with newly diagnosed GM, five men and six women were included. Six (55%) patients had a long history of hypertension, three patients (27%) had obesity, and two patients (18%) had coronary heart disease. All the studied patients did not have a history of VTE. According to MRI data, in 27% (*n* = 3) of the patients, the tumor size was more than 5 cm, which is an independent risk factor for cancer-associated VTE. At the same time, 73% (*n* = 8) of the patients had signs of brain dislocation syndrome. However, the initial functional status defined by a Karnofsky Performance Status (KPS) was quite good (pre-surgical KPS ≥ 70%). Based on the histological findings obtained within 72 h of surgery, 27% (*n* = 3) of the patients had low-grade GM, and glioblastoma in 73% (*n* = 8) of cases was confirmed.

### Laboratory Results

Patients with GM had significantly higher white blood cell (WBC) and neutrophils counts than in the control group (8.0 [5.6; 10.3] × 10^9^/L vs. 5.9 [5.2; 6.6] × 10^9^/L, *p* = 0.016 and 5.8 [5.1; 9.9] × 10^9^/L vs. 3.2 [2.8; 3.9] × 10^9^/L, *p* = 0.002, respectively). The platelet count did not differ between the groups (224 [165; 278] × 10^9^/L in the patients with GM vs. 227 [187; 255] × 10^9^/L in the control group, *p* = 0.81).

Among the analyzed coagulation parameters [prothrombin time (PT), activated partial thromboplastin time (APTT), D-dimer, and fibrinogen levels], only D-dimer was significantly increased in the patients with GM compared with the control group (0.46 [0.38; 1.85] μg/ml FEU vs. 0.36 [0.27; 0.40] μg/ml FEU, *p* = 0.03, respectively). Levels of C-reactive protein (CRP) did not differ between the groups (2.5 [0.3–4.8] g/L vs. 1.4 [0.3–4.0] g/L, *p* > 0.05).

As shown in Table 1, the relative abundance of the CD41 + EVs and CD105 + EVs were significantly higher in the GM group than in the control group.

**TABLE 1:** Cell-derived extracellular vesicles (EVs) ratio in patients with glioma and controls.

	Glioma group (*n* = 11) Me [IQR 25th; 75th]	control group (*n* = 10) Me [IQR 25th; 75th]	*P*
CD45 + EVs, %	6.0 [5.5; 6.8]	8.3 [7.5; 9.6]	0.07
CD235 + EVs, %	4.4 [3.4; 5.9]	6.2 [5.3; 6.3]	0.61
CD41 + EVs, %	44.3 [40.5; 52.4]	27.2 [22.9; 31.0]	0.002**
CD105 + EVs, %	5.4 [4.8; 7.8]	1.9 [1.5; 2.8]	0.0003****

p* < 0.05; ** < 0.01; *** < 0.005, and **** < 0.001.

No significant differences in TGT data in the GM group were found compared with the control group, but Peakthr. and VI had a tendency to increase in the patients with GM (271.0 [225.4; 288.7] vs. 223.0 [197.5; 249.5] nmol, *p* = 0.06 and 95.7 [64.9; 102.3] vs. 51.3 [42.4; 67.9] nmol/min, *p* = 0.06, respectively).

**FIGURE 1:**
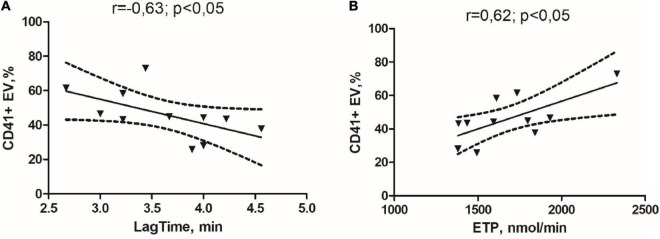
Correlation links between the initiation time of coagulation in the study of thrombin generation test (TGT) and the percentage of the CD41 + EVs **(A)** and endogenous thrombin potential in the study of TGT and percentage of the CD41 + EVs in the glioma group **(B)**.

As shown in Figure 1, in the GM group, we obtained a significant negative correlation between circulating relative abundance of CD41 + EVs and Lag time (Figure 1A) and a significant positive correlation between relative abundance of CD41 + EVs and ETP in the study of TGT (Figure 1B).

Within the observed group of patients, two patients with GM (18%) had deep vein thrombosis in a short period of postoperative follow-up (14 days*).* These patients had increased D-dimer (>5 μg/ml FEU), VI (>95 nmol/min), and thrombocytopenia < 100 × 10^9^/L and both of them had a tumor size of more than 5 cm.

## Discussion

Venous thromboembolism is a common complication in patients with primary GM (about 20%) (18). Clinical and laboratory parameters have been determined as risk factors for VTE in GM patients with numerous attempts to develop the thrombosis risk scales (2). Risk factors in brain tumors can be classified as the patient-, treatment-, and cancer-related risk factors (18). Among the risk factors associated with the patient, the most significant are age and the degree of functional activity decrease, most often associated with paresis (19). Considering the pre-surgical KPS ≥ 70% and the average age of less than 65 years, our study group had low patient-related thrombosis risk factors. The patients did not differ in treatment-related risk factors: all underwent total tumor resection without a preliminary biopsy, and no chemotherapy or radiation therapy was started during a short follow-up period.

It was shown that the grade of the brain tumor was decisive. According to the Vienna Cancer and Thrombosis Study, the cumulative VTE risk after 2 years of follow-up in a cohort of patients with different primary brain tumors was 14.8%, with 89% of those having a high-grade GM (20). We consistently included in the study only patients with a high probability of glioblastoma according to MRI data, but high-grade GM was confirmed by histological examination only in 73% (*n* = 9) of cases. Two of these patients had deep vein thrombosis in a short period of postoperative follow-up (14 days). This fact stresses the importance of the identification of special, tumor-associated factors that play a crucial role in thrombosis risk and require further investigation as well as a search for laboratory markers that reflect the mechanism of thrombosis development and the degree of hemostasis disturbance.

Riedl and Ay’s recent paper suggested the use of some simple laboratory markers for thrombosis risk stratification: high WBCs count, low platelet count with high soluble P-selectin levels as markers of activated platelet, and increased coagulation factor VIII activity in combination with increased D-dimer levels (18). According to our results, the GM group had a slightly higher number of WBCs compared with the control group due to the increased number of neutrophils but remained within the reference values. Another research study demonstrated the potential role of neutrophils in thrombosis development, where microvascular thrombosis was based on the fibrin generation inside blood vessels and triggered by neutrophils-platelets interaction, which resulted in the release of neutrophil extracellular traps (21). Another significant thrombosis risk factor in brain tumors is thrombocytopenia, while in most other tumors, an increased platelet count has been found. The reason for low platelet count might be platelet aggregation caused by podoplanin binding to platelet C-type lectin receptor 2 (22). In the present study, thrombocytopenia < 100 × 10^9^/L was detected in patients with high-grade GM and confirmed VTE, which is consistent with the mentioned studies.

In our pilot study, the GM group differed from the control group by a higher D-dimer level (*p* = 0.03). To assess the more detail, we applied TGT. No significant differences were found between the groups. However, in the GM patients, Peakthr. and VI had a tendency to be increased (*P* = 0.06), along with raised D-dimer. The shift toward a hypercoagulable state in the brain tumour group has been confirmed. TF as the primary activator of coagulation plays a central role in the concept that the hemostatic system and tumor growth create a vicious circle (23). Since the EVs carry a phospholipid membrane—a site for the assembly of coagulation factors—and can retain TF, their procoagulant potential and role in the development of VTE are being actively studied (24). Furthermore, tumor cell-derived EVs exhibit strong procoagulant activity *in vitro* and *in vivo* (25). Results obtained by Sartori et al. showed that baseline levels of glial and endothelial EVs were significantly higher in patients with glioblastoma than in the control group and significantly increased after surgery (1 week, 1, 4, and 7 months). The number of TF-bearing EVs was significantly higher in patients who developed VTE compared with those who did not (*p* = 0.04) (3). However, a recent review article reported that an association between EV TF activity and VTE was found only in patients with pancreatic cancer as well as mortality in patients with cancer (26). Our results showed that in the group of patients with GM, the relative abundance of platelet- and endothelial-derived EVs was significantly increased compared with donors. Increased EV production is probably associated with platelet activation due to interaction with such physiological platelet agonists as thrombin and collagen. Our previous EV study showed that most platelet-derived EVs are separated from activated cells (platelets) and, respectively, carry the activation marker CD62P on their surface (27). Thus, on the one hand, the increased number of circulating EVs leads to an increase in TG, but the opposite situation is also possible because thrombin itself is a powerful platelet activator with an increasing platelet-derived EVs number.

In addition, a correlation was observed between the increase in the relative abundance of the CD41 + EVs and raised procoagulant activity defined by TGT. The correlation found is not strong and requires confirmation in a larger sample. However, the results from the “Vienna Cancer and Thrombosis Study” confirmed the predictive role of TGT in assessing the risk of VTE in patients with malignant neoplasms of various localizations (*n* = 1033, hazard ratio [*HR*] = 2.1 (95% *CI*, 1.3–3.3, *p* = 0.002) (8). It seems relevant to evaluate the TG ability in terms of hypercoagulable state assessment and thrombosis prediction in the glioma group.

Our study is limited by the small sample size and lack of EVs procoagulant state assessment.

## Conclusion

Our primary results demonstrate the potential ability to use the relative abundance of platelet-derived EVs, TGT data, D-dimer level, and platelet counts as laboratory biomarkers for VTE risk stratification in patients with GM. Further prospective studies using a large sample are required to clarify the specifics of glioma patients’ VTE risk factors, including reproducible simple laboratory tests for thrombosis risk stratification in these difficult to manage patients.

## Data Availability Statement

The raw data supporting the conclusions of this article will be made available by the authors, without undue reservation.

## Ethics Statement

The studies involving human participants were reviewed and approved by Ethics Committee Federal State Budgetary Institution “Almazov National Medical Research Center” of the Ministry of Health of the Russian Federation. Extract from the minutes of the meeting No. 0603-21 of the local ethics committee No. 03-21 dated March 15, 2021. The patients/participants provided their written informed consent to participate in this study.

## Author Contributions

OM, OS, YZ, and MS had revised the manuscript critically for important intellectual content. YZ, OM, and EZ performed all laboratory studies of the collected biomaterial. KP, MT, AP, and MS participated in the clinical diagnostic and treatment of patients with glioma. All authors made a substantial contributions to the design of the work, carried out the collection and analysis of data for the work, participated in drafting a work, and agreed to be accountable for all aspects of the work in ensuring that questions related to the accuracy or integrity of any part of the work are appropriately investigated and resolved.

## Conflict of Interest

The authors declare that the research was conducted in the absence of any commercial or financial relationships that could be construed as a potential conflict of interest.

## Publisher’s Note

All claims expressed in this article are solely those of the authors and do not necessarily represent those of their affiliated organizations, or those of the publisher, the editors and the reviewers. Any product that may be evaluated in this article, or claim that may be made by its manufacturer, is not guaranteed or endorsed by the publisher.
