# Supplementary material for: Circulating Small Extracellular Vesicles Profiling and Thrombin Generation as Potential Markers of Thrombotic Risk in Glioma Patients
Source: Front Cardiovasc Med. 2022 Jun 23;9:789937. doi: 10.3389/fcvm.2022.789937 (PMC9259782; doi:10.3389/fcvm.2022.789937)
Supplement: Supplementary file 1 [file Data_Sheet_1.docx]

# Supplementary materials


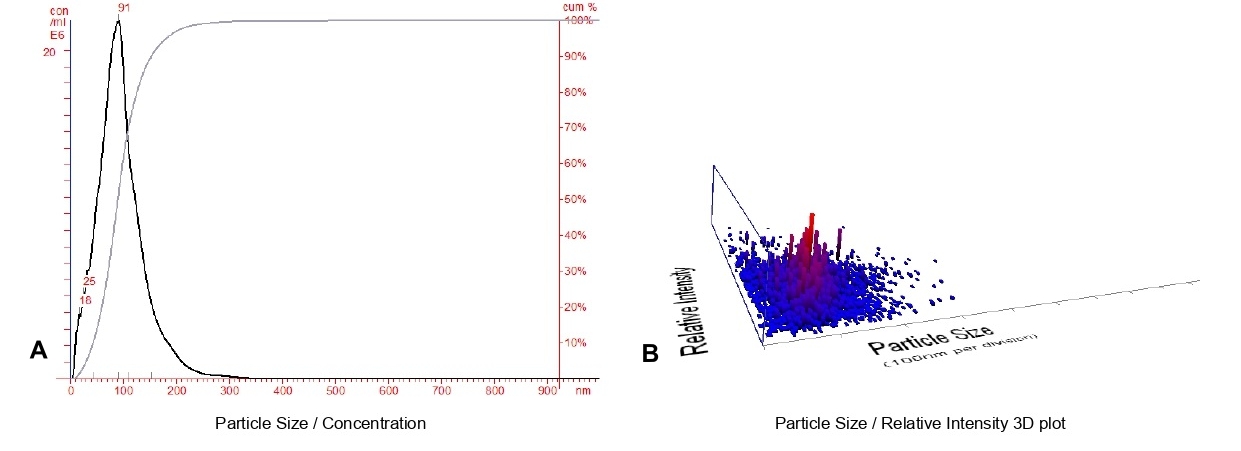


**Supplemental Figure S1.** **Represents example size distribution from nanoparticle tracking analysis (NTA) measurements of extracellular vesicles (EVs) the sample of patient’s №1. A) Mean Size and Size Distribution from NTA measurements EVs the sample of patient’s №1; B) Relative intensity 3D plot represents of the size distribution profile of EVs**

The size, homogeneity, and concentration of EVs in the obtained preparations were assessed using an nanoparticle tracking analysis (NTA) NanoSight® LM10 (Malvern Instruments,UK) analyzer equipped with a blue laser (45 mW at 488 nm) and a C11440-5B camera (Hamamatsuphotonics K. K., Japan). The NTA 2.3 software was used to record and analyze the obtained results. When analyzing records lasting 60 seconds, the following parameters were evaluated: the average hydrodynamic diameter (nm) and the concentration of microvesicles in suspension (particles/ml). As a result, in the presented sample of patient’s №1 mean size observed EVs was 91±45 nm (mean ± SD).


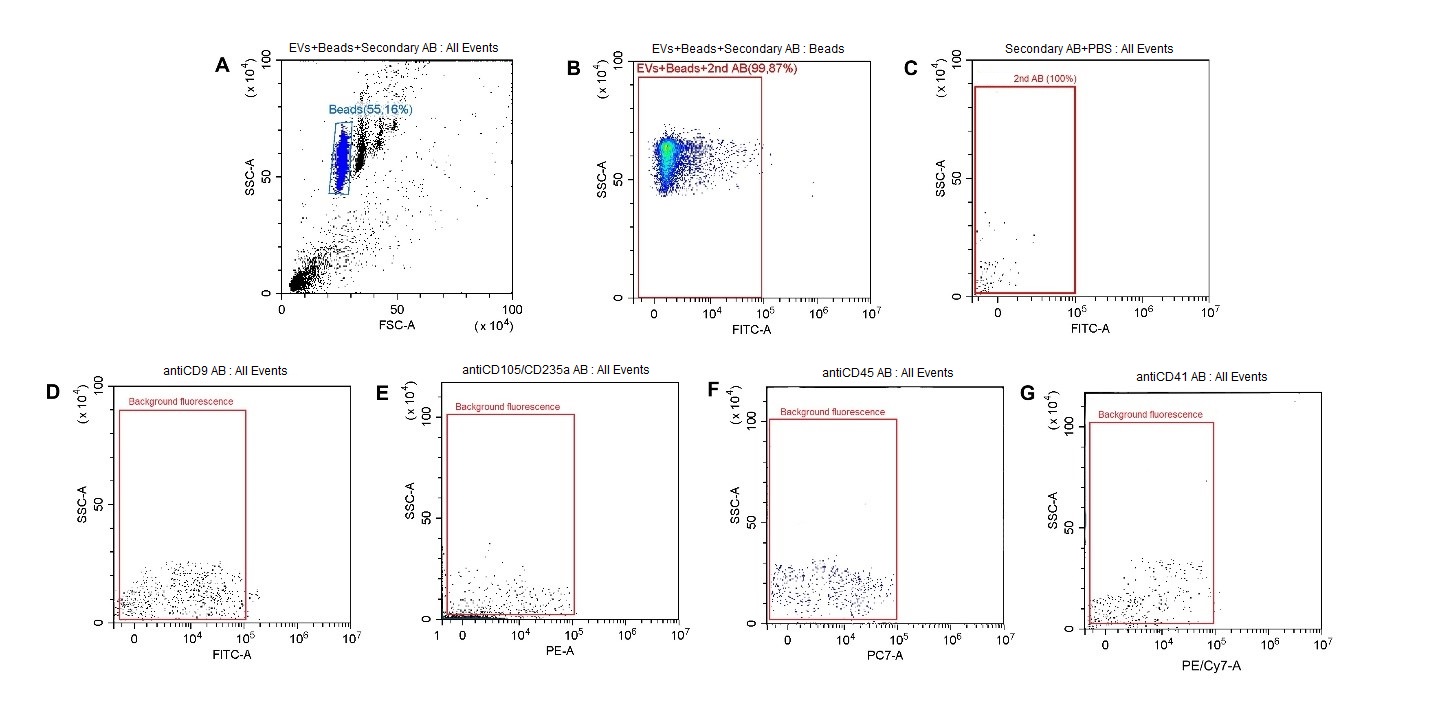


**Supplemental Figure S2. Represents control scatter plots performed using flow cytometer CytoFlex B4-R2-V2 to exclude non-specific binding and background fluorescence A) Control dot plot from all events containing EVs-beads complex stained with secondary antibodies (against anti-CD9 primary antibodies); B) Control dot plot from beads gate containing EVs-beads complex stained with secondary antibodies; C) Control dot plot containing PBS stained with secondary antibodies; D) Control dot plot containing PBS stained with anti-CD9 primary and secondary FITC antibodies; E) Control dot plot containing PBS stained with CD235a-PE and CD105-PE antibodies; F) Control dot plot containing PBS stained with CD45-PC7 antibodies; G) Control dot plot containing PBS stained with CD41-PE/Cy7 antibodies.**

Control samples were stained and analysed on the Cytoflex B4-R2-V2 using the same instrument settings as for the EVs samples. The gates for positive events were set according to the controls to ensure that no target events were cut off.


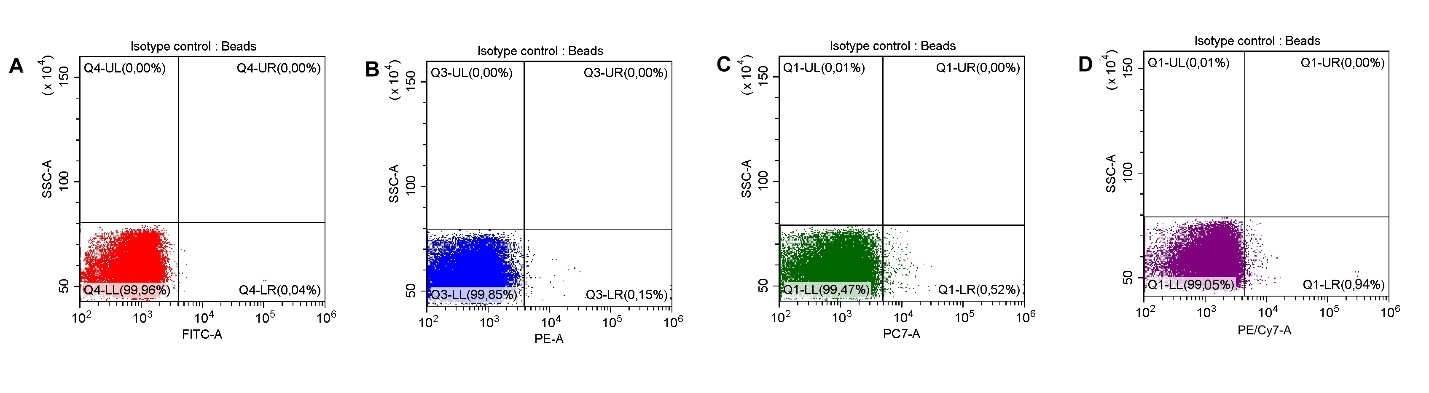


**Supplemental Figure S3. Represents isotype control dot plots performed using flow cytometer CytoFlex B4-R2-V2 A)** **FITC-antiMouse IgG1 isotype control; B) PE-antiMouse IgG1 isotype control; C) PC7-antiMouse IgG1 isotype control; D) PE/Cy7-antiMouse IgG1 isotype control.**


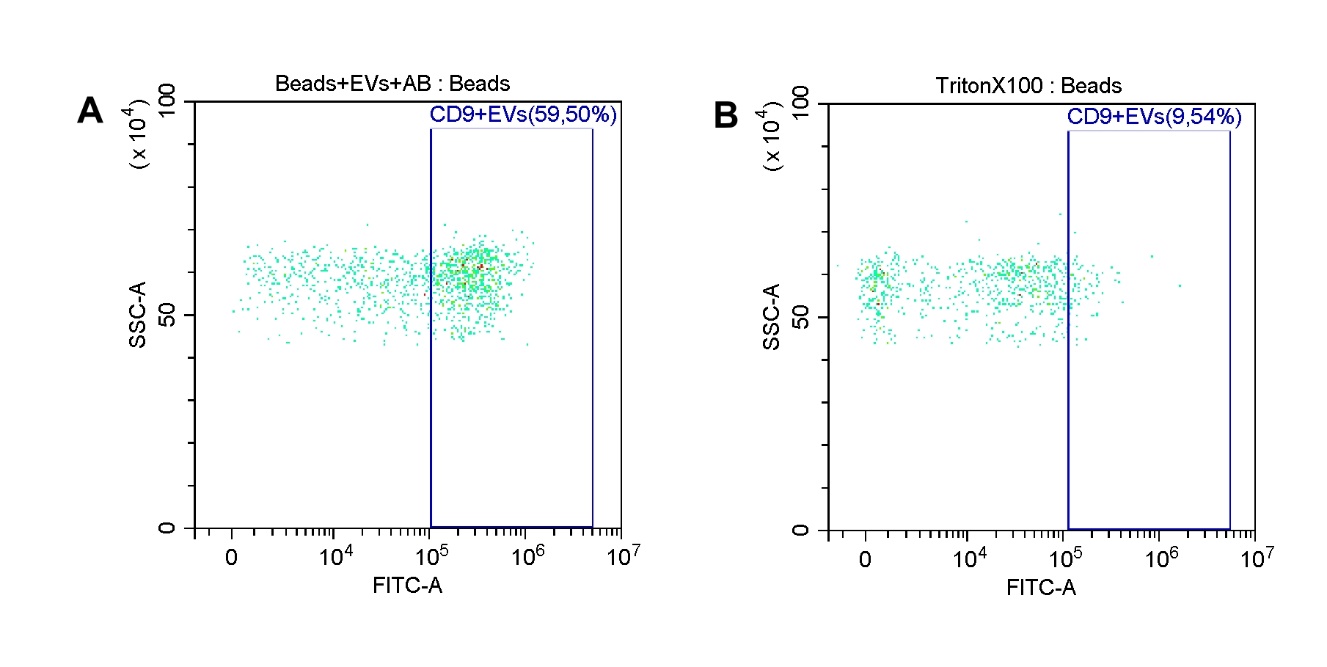


**Supplemental Figure S4. Detergent control of the EVs performed using flow cytometer CytoFlex B4-R2-V2. A) Represents results before Triton X100 treatment B) After Triton X100 treatment.**

To prove that samples contained EVs, additional controls with detergent treatment were performed. EVs decorating beads population was stained with anti-CD9-antibodies and phenotyped after incubation at room temperature for 10 min with equal volume of either PBS or 2% Triton X100 in PBS. Pictures before and after Triton X100 were normalized to experiment acquisition time.


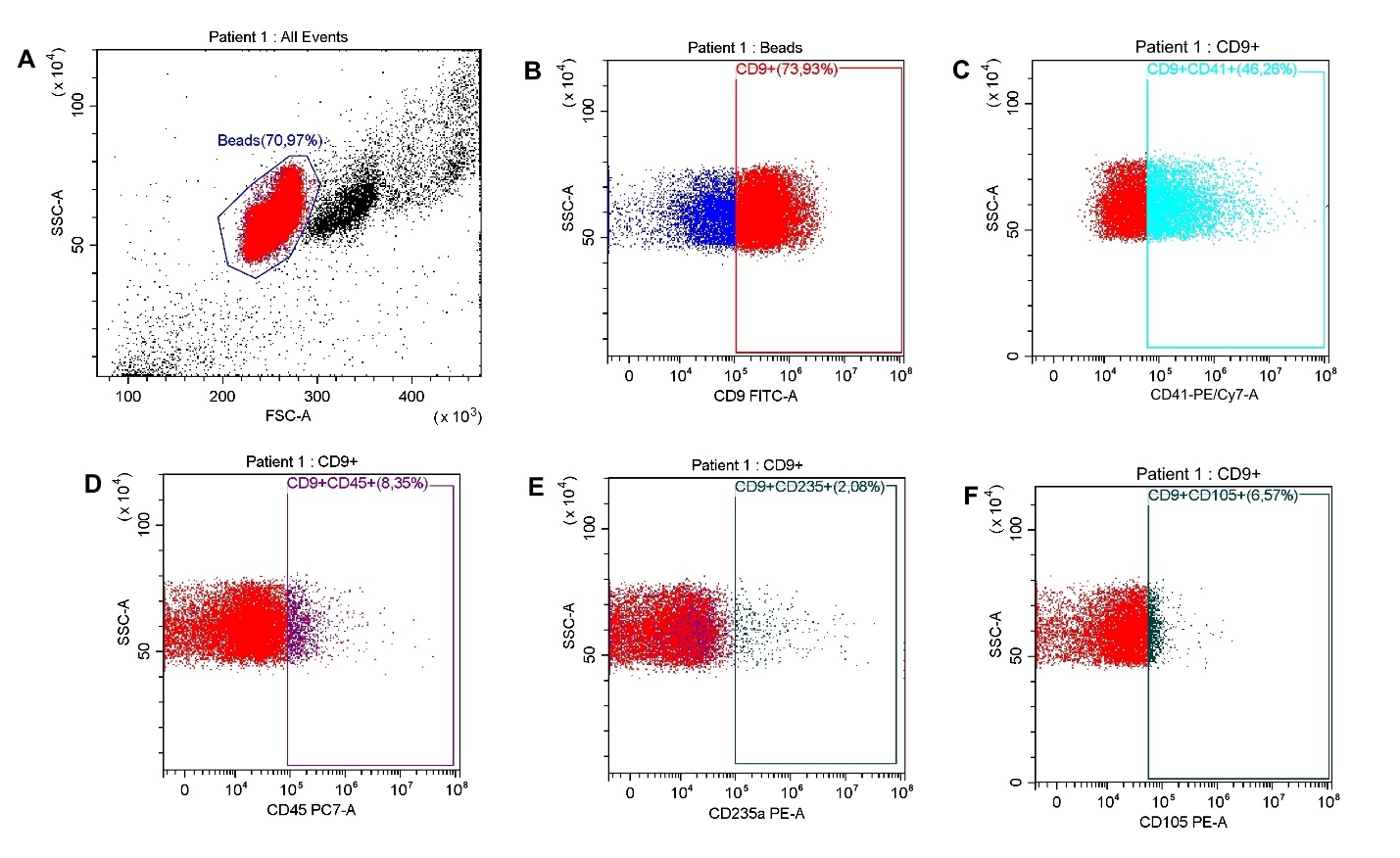


**Supplemental Figure S5. Representative results of patient’s sample dot plots showing the percentage of positive populations CD41 (PE/Cy7), CD45 (PC7), CD235a (PE) and CD105 (PE) among CD9+ events (cell-derived EVs). A) Dot plot of side scatter vs. forward scatter intensity EVs decorating beads population; B) Corresponding SSC vs. CD9-FITC-A dot plot; C) Corresponding SSC vs. CD41-PE/Cy7 dot plot; D) Corresponding SSC vs. CD45-PC7 dot plot; E) Corresponding SSC vs. CD235a-PE dot plot; F) Corresponding SSC vs. CD105-PE dot plot.**

Two testing tubes were stained: 1st — CD9 (AlexaFluor488), CD235a (PE), CD 45 (PC7), 2nd - CD9 (AlexaFluor488), CD41 (PE/Cy7) и CD105 (PE). We had got 20000 events from the gated first population. Samples were run at constant flow rate below 120 μL/min. At first Beads area was gated (latex beads to which exosomes were adsorbed) using forward scatter (FSC) and side scatter (SSC) gating strategies Then CD9+ are was selected (from 70 to 90 per cent Beads carried CD9+) from the Beads gate. In the last step, areas were gated with cell-markers positive events (CD41+, CD45+, CD105+, CD235a+) from the CD9+ gate. Dot plots were used to show the percentage of positive populations CD45 (PC7), CD235a (PE), CD41 (PE/Cy7), and CD105 (PE) among CD9+ events (cell-derived EVs). The gates for positive events were set according to the controls.


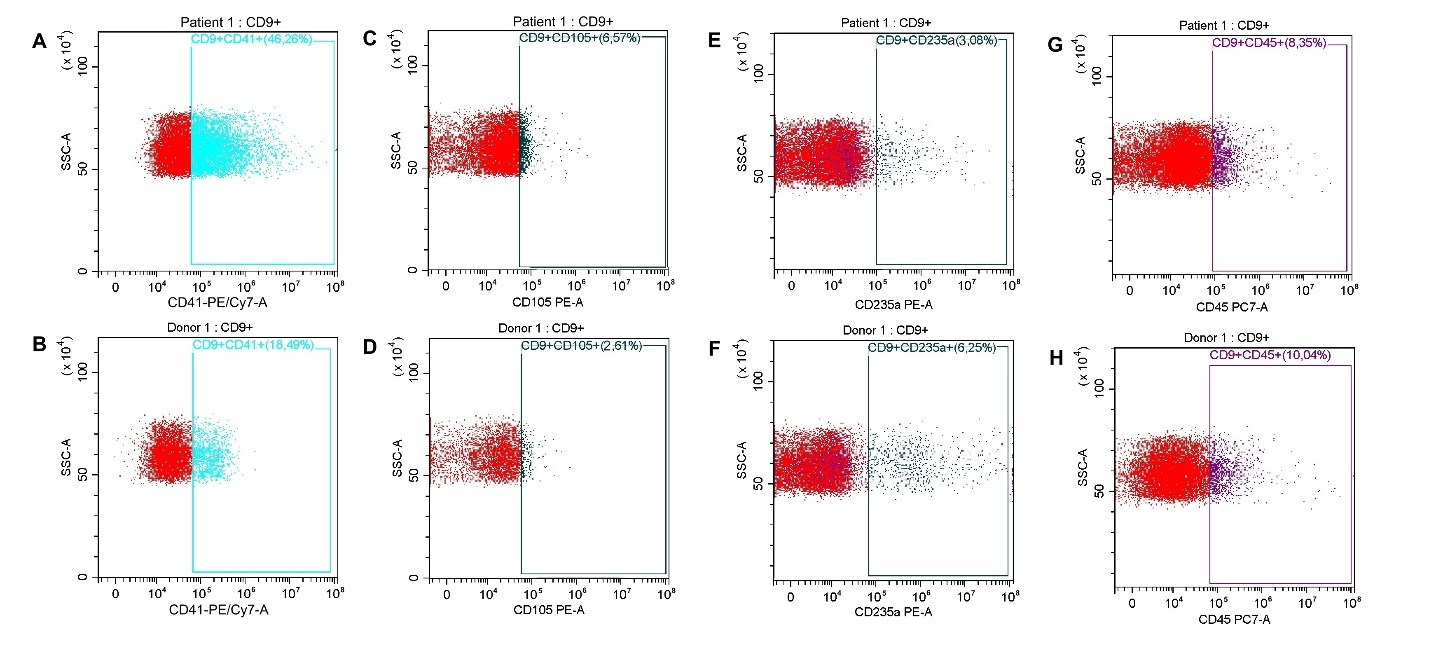


**Supplemental Figure S6. Representative results of dot plots of donor’s and patient’s samples comparing. A) Corresponding count vs. CD41-PE/Cy7 dot plot of patient’s sample; B) Corresponding count vs. CD41-PE/Cy7 dot plot of donor’s sample; C) Corresponding count vs. CD105-PE dot plot of patient’s sample; D) Corresponding count vs. CD105-PE dot plot of donor’s sample; E) Corresponding count vs. CD235a-PE dot plot of patient’s sample; F) Corresponding count vs. CD235a-PE dot plot of donor’s sample; G) Corresponding count vs. CD45-PC7 dot plot of patient’s sample; H) Corresponding count vs. CD45-PC7 dot plot of donor’s sample.**
